# A biologically based neural network model for decision making

**DOI:** 10.1186/1471-2202-16-S1-P146

**Published:** 2015-12-18

**Authors:** Azadeh Hassanejad Nazir, Hans Liljenstrom

**Affiliations:** 1 Department of Energy and Technology, SLU, Uppsala, SE-75007, Sweden; 2 Agora for Biosystems, Sigtuna, SE-19322, Sweden

## 

**Figure 1:**
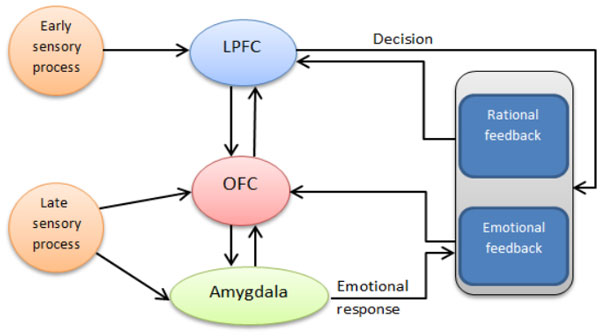
**Interactions of the main neural structures in the decision making process: Amygdala, orbitofrontal cortex (OFC) and lateral prefrontal cortex (LPFC)**. Inputs from sensory modalities and from rational and emotional feedback.

**Figure 2:**
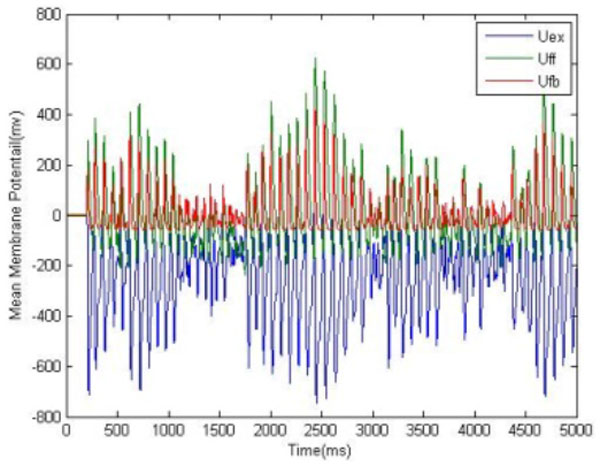
**Activity of a cell assembly representing one of possible options**. Red and green traces show the activity of the inhibitory neurons and the blue one the activity of all excitatory neurons.

We present a neural network model, describing an adaptive decision making process (DM), under varying internal and external contexts [1]. The model includes the three most crucial structures in both emotional and rational aspects of DM: amygdala, orbitofrontal cortex (OFC) and lateral prefrontal cortex (LPFC) [2-4]. Neural activities in these structures represent emotional attitude, expectancy value, and rules towards the options (Figure 1). The DM is modeled at a level of mesoscopic neurodynamics, using biologically inspired neural networks [5]. Rational and emotional associations are encoded with cell assembly oscillations in all three structures, determining the value of an option, *V*(*opt*), as a product of the number of activated cells and the mean frequency and amplitude of their oscillations (Figure 2). A decision is based on the competition among stored patterns, using cosine similarity of the frequency vectors $\vec{\text{f}}$. The option with highest value will win the competition in each system:

$$\text{V}\left(\text{opt}\right)|{\text{s}}_{\text{opt}}|.\left\langle \vec{{\text{f}}_{\text{opt}}},\vec{{\text{A}}_{\text{opt}}}\right\rangle =|{\text{s}}_{\text{opt}}|.\left\langle \vec{{\text{f}}_{\text{opt}}},\vec{{\text{A}}_{\text{opt}}}\right\rangle ,\;\forall \;\text{opt}=1,...,\text{n}$$

The emotional system is goal directed on the predicted value of the options. Emotional memory of various options are formed and stored in the amygdala. A prediction signal is generated in OFC and an emotional response is measured as a level of satisfaction.

LPFC is a pivotal structure in the rational analysis and is based on habitual DM. Declarative and procedural memories, as the main components of this system are activated by external stimuli. The bidirectional interactions between maintained rules in procedural memory, rational attitudes towards different options, and stored factual information in declarative memory, lead to the selection of an option as a rational decision.

The integration of emotional and rational activities results in a final decision. When an action is executed as a result of a decision its experienced value is compared with the expected one, and stored in memory. Depending on the sign and the magnitude of the prediction error (expected value - real value), the stored emotional and rational attitude might be updated.

The experience of our decisions/choices are learnt and may influence future decisions. For any particular input signal, the final decision could shift, depending on internal and external context. Large delayed rewards have a lower value, compared to small immediate rewards. This fact can be included with the help of a hyperbolic discounting function, which models the exponential reduction of rewards in terms of time.
